# Use of Granulocyte Transfusions in the Management of Severe Infections Among Children with Neutropenia

**DOI:** 10.3390/jpm14111107

**Published:** 2024-11-15

**Authors:** Gabriela Mielecka-Jarmocik, Katarzyna Szymbor, Walentyna Balwierz, Szymon Skoczeń, Marta Leń, Kinga Kania, Katarzyna Pawińska-Wąsikowska

**Affiliations:** 1 Department of Pediatric Oncology and Hematology, Institute of Pediatrics, Jagiellonian University Medical College, 30-663 Krakow, Poland; kszymbor@usdk.pl (K.S.); walentyna.balwierz@uj.edu.pl (W.B.); szymon.skoczen@uj.edu.pl (S.S.); 2 Department of Pediatric Oncology and Hematology, University Children Hospital of Krakow, 30-663 Krakow, Poland; 3 Department of Serology and Transfusion at the University Children’s Hospital of Krakow, 30-663 Krakow, Poland; mlen@usdk.pl (M.L.); kkania@usdk.pl (K.K.)

**Keywords:** granulocyte transfusions, neutropenia, children, haematologic disease, malignancy

## Abstract

**Background/Objectives:** Infections remain the leading cause of mortality among neutropenic patients with haematologic malignancies, making effective infection management crucial. Achieving a sufficient neutrophil count is essential for the elimination of pathogens. Granulocyte concentrate (GC) can be a treatment option for neutropenic patients with severe infections. This study aimed to evaluate the efficacy, safety, and impact on survival of GC transfusions in neutropenic children with severe infections treated over the past 13 years in a single centre. **Methods:** The retrospective study analysed clinical data from 60 children (median age 9.5 years) who received GC transfusions at our centre. Granulocytes were collected by apheresis from donors stimulated with granulocyte colony-stimulating factor. The majority of the patients (70%) were diagnosed with acute leukaemia. The main indications for GC were severe pneumonia (45%) and bacterial sepsis (38.33%). **Results:** The patients received 1 to 29 GC transfusions for 1 to 70 days, with a median time of administration of 3 days. Neutrophil counts increased to >1000/µL within a median of 5 days. GCs were well tolerated by most patients. One patient presented symptoms of anaphylaxis, the other acute lung injury related to transfusions, and alloimmunisation was reported in one patient. Of the patients analysed, 78.33% survived the infection that justified GC administration. We did not observe significant differences in survival depending on the aetiology of the infection. **Conclusions:** Based on our research, GC appears to be a beneficiary for neutropenic children with severe infections and reduces infection mortality rates. However, further well-designed randomised trials are needed to define its role in this setting.

## 1. Introduction

The last three decades have brought about significant improvement in the treatment outcomes of children with malignancy. This undoubted success has been achieved not only through the implementation of an individualised approach to therapy based on risk factors and the introduction of targeted therapies and biologics, but also through the development of supportive treatment [1]. Infections are still the leading cause of mortality among neutropenic patients with haematologic malignancies and those who have undergone haematopoietic stem cell transplantation; therefore, infection management remains crucial to improve survival [2,3,4]. This implies the use of broad-spectrum antibiotics, antifungal and antiviral drugs, haematopoietic colony-stimulating factors, specific and nonspecific immunoglobulin, and other blood products [5,6].

Despite the variety of supportive treatment methods, it appears that the critical factor for eradicating an infection is to obtain a sufficient neutrophil count initially in the circulating blood and finally in the infected tissues [7,8,9]. Unfortunately, very often, after intensive chemotherapy, radiotherapy, or due to malignant clones in the bone marrow, it is not possible to achieve haematopoietic regeneration in the expected time. Granulocyte concentrate (GC) is one of the blood products that can be a treatment option for patients with neutropenia and severe infection. The justification for the use of GC has remained the subject of research for more than 50 years without clear conclusions and recommendations for its use [9,10,11]. During this time, the technologies for obtaining granulocytes from matched donors have changed many times. The experience of different centres varies, which is reflected in the highly heterogeneous frequency of GC use and the lack of strict recommendations in this area. Additionally, the 2015 and 2016 Cochrane systematic reviews covering the prophylactic and therapeutic use of GC, respectively, do not provide conclusive statements, mainly due to the low quality of the evidence [12,13]. At the same time, infections, increasingly caused by pathogens with an expanded spectrum of resistance, remain a permanent problem for all patients who are profoundly neutropenic as a result of their underlying disease or therapy [14]. However, there are many controversies regarding the use of GC and its definitive role as a supportive care measure has not yet been determined. There is no standard approach to GC administration in neutropenic children, as the number of studies is limited, and the indications, optimal dose, and duration of therapy vary between them [7,9,10,12,15].

The aim of our study was to determine the efficacy, safety, and impact on survival of granulocyte concentrate transfusion in neutropenic children with severe infections treated in our centre in the last 13 years.

## 2. Materials and Methods

### 2.1. Patient Populations

We retrospectively analysed the medical records of children treated in the Department of Pediatric Oncology and Hematology, Institute of Pediatrics, Jagiellonian University Medical College between January 2010 and December 2023.

Sixty-five neutropenic patients were treated with GC in this period; however, eventually sixty of them were included in the study. Five patients were excluded due to the lack of sufficient medical data. The criteria for initiating GC transfusion in the analysed population included: leukopenia with neutropenia (particularly prolonged), resulting from the underlying bone marrow disease or oncological treatment, clinical and/or laboratory signs of infection (fever, deterioration of general condition, increased levels of inflammatory markers) and availability of granulocyte donors. Microbiological identification of the etiological agent was not required.

Basic data such as the initial diagnosis, demographic characteristics of patients, and indication of GC transfusion such as the type and cause of infection were collected from the medical database. The study protocol has been carried out in accordance with The Ethics Code of the World Medical Association (Declaration of Helsinki) for experiments involving humans and approved by the Ethics Committee of Jagiellonian University (protocol code—118.6120.23.2023, date of approval: 31 May 2023).

### 2.2. Donors, Preparation, and Transfusions of GC

GC was obtained by apheresis from a single voluntary donor using cell separators with hydroxyethylated starch (HES) as an agent to accelerate erythrocyte sedimentation and sodium citrate as an anticoagulant.

Prior to donation, the volunteers underwent preliminary qualification at the Blood Donor Centre in accordance with the established protocols, including screening for HBV, HCV, HIV, and syphilis infections. The procurement of an adequate granulocyte yield in the preparation was achievable only following appropriate donor stimulation. To this end, after the successful completion of the initial qualification stage, the donors were referred to the Department of Pediatric Oncology and Hematology, where based on cross-matching results, they were administered a 480 μg subcutaneous dose of recombinant granulocyte colony-stimulating factor:G-CSF, Filgrastim, Neupogen(Amgen Inc., Thousand Oaks, CA, USA).

All the donors provided written informed consent for G-CSF stimulation and granulocyte apheresis. On the following day, approximately 12 h (ranging from 8 to 16 h) post-G-CSF administration, the donors were admitted to the Blood Donor Centre for leukapheresis. The goal was to obtain 1.2 × 10^10^ granulocytes in preparation to ensure a dose of >1.5–2 × 10^8^/kg body weight in children and adolescents, and above 1 × 10^9^/kg body weight in neonates [9,10,12,15]. GCs contain a residual amount of other white blood cells, erythrocytes, and platelets [16]. Due to the amounts of erythrocytes in GCs (above 2 × 10^10^/unit) every transfusion was preceded with a serological compatibility test. ABO compatibility and the correct result of the cross-match were a necessary condition for the initiation of donor stimulation. All the GCs were irradiated to prevent transfusion-associated graft versus host disease (TA-GvHD). The final product was delivered to the Department of Pediatric Oncology and Hematology and transfused to the patient as soon as possible after preparatory procedures. In a situation where it was not possible or due to the weight of the recipient, the GC was divided into parts and the GCs were stored at 20–24 degrees Celsius without agitation for up to 24 h. Premedication with paracetamol, hydrocortisone, and clemastine was administered to each patient prior to transfusion. During GC transfusion, patients’ vital signs were monitored to detect acute transfusion-related toxicity.

### 2.3. Outcome Measures

To analyse the clinical benefits of GC transfusion, we evaluated the clinical and laboratory responses of patients to GC therapy. The volume of GC, number of administrations, number of granulocytes in the product, and duration of its use were taken into account. The onset of infection was defined as the day on which an increase in inflammatory markers (previously low or undetectable) was first noted or the first episode of fever was documented.

Based on generally accepted definitions, when analysing GC quality, we took a granulocyte count of ≥1.5 × 10^8^ per kilogramme of body weight as a sufficient-dose granulocyte transfusion and 6 × 10^8^/kg as a high-dose granulocyte transfusion [10].

The type of infection and biomarkers of its severity, such as C reactive protein (CRP), procalcitonin (PCT), and hospitalisation in the intensive care unit (ICU) were evaluated. We also analysed the use of other supportive care measures (antibiotics, antifungals or antivirals, and G-CSF). The outcome of therapy was evaluated by the patient’s survival and the time to recovery from infection.

### 2.4. Statistical Analysis

Descriptive statistics for continuous variables were presented as the mean with standard deviation [SD] or median with interquartile range [IQR] for normally and non-normally distributed data, respectively. Categorical variables were shown as the number of cases and percentage values. The significance level of 0.05 was used in all the statistical tests. Statistical analysis was performed using Microsoft Excel for Windows and Statistica, version 14.

## 3. Results

Between January 2010 and December 2023, 65 children and adolescents with neutropenia were treated with GC for severe infections. A total of 60 patients were eventually included in the study: 29 girls (47%) and 33 boys (53%). The median age of the patients was 9.5 years (range: 4 months–19 years).

The majority of the GC recipients were diagnosed with acute myeloid leukaemia (n = 22, 36.7%) as the primary diagnosis. Most of the patients were in the first complete remission (CR) when GC was administered, 13 children (21.7%) were treated for relapse, 5 (8.3%) for secondary cancer, and 4 (6.7%) had a haematologic (noncancerous) diagnosis. The characteristics of the patients are presented in Table 1.

**Figure 1:**
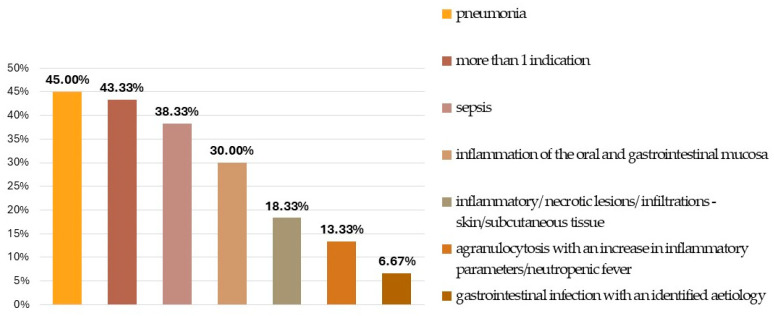
Infectious indications for GC treatment.

Bacterial pneumonia and sepsis were the predominant indications for GC transfusion; in addition, oral mucositis, gastrointestinal infection, and inflammatory/necrotic/infiltrative lesions located on the skin or subcutaneous tissue also had a non-negligible frequency. Twenty-six patients (43.3%) had more than one infection foci (Figure 1).

The bacterial aetiology of the infections predominated (n = 23; 38.3%) and the material used for microbiological identification was mainly blood culture. In 25% of the patients, the aetiological factor could not be identified. Among the identified bacterial strains, we observed antibiotic-resistant variants, including Enterococcus faecium HLGR (high-level gentamicin-resistant), Klebsiella pneumoniae ESBL (extended-spectrum beta-lactamase), coagulase-negative staphylococci MRCNS/MLSB (methicillin-resistant coagulase-negative staphylococci and macrolide–lincosamide–streptogramin B-resistant), Escherichia coli ESBL, and Staphylococcus capitis MRCNS/MLSB. The details of the infections, together with the highest values of the inflammatory markers observed, are summarised in Table 2 and Table 3.

**Figure 2:**
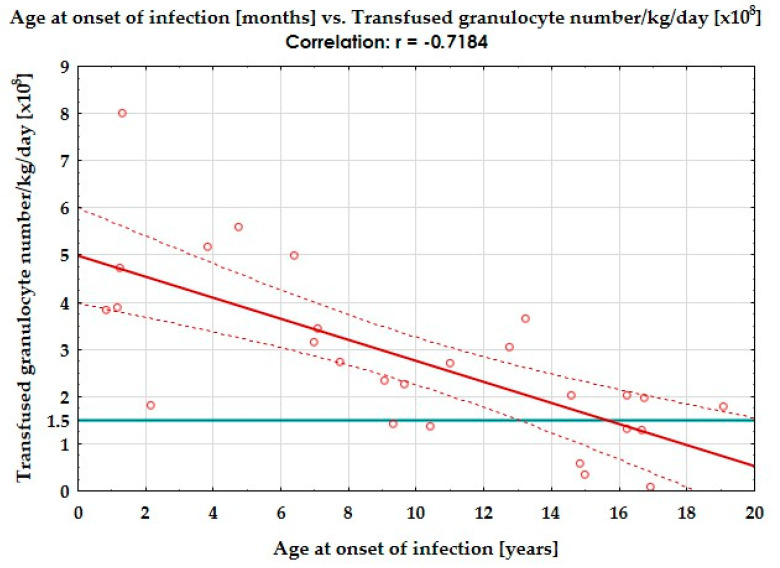
The correlation between the patient’s age and the received number of granulocytes per kilogramme of body weight per day. Continuous red line—the graphical representation of negative correlation; dashed orange lines—the 95% confidence intervals for the correlation line; orange dots—the single records; turquoise solid line - the border above which the number of granulocytes in GC was sufficient.

In the analysed group, the median time from the symptoms of infection to the first GC transfusion was 5 days (IQR 3–9.0). The patients underwent 1 to 29 GC transfusions over a period of 1 to 70 days. The median time of GC administration was 3 days (IQR = 1–5.5 days), and an increase in neutrophil count to >1000/μL was observed with a median of 5 days (IQR = 2–9). Data on the granulocyte content in the transfused concentrate were obtained for 28 participants (143 transfusions). GCs with a sufficient granulocyte count (≥1.5 × 10^8^ per kilogramme of body weight) represented 76.2% of all the GCs, while the high-dose concentrate (6 × 10^8^/kg) represented only 24.5%. However, these values differed significantly by child age and weight (≤25 kg vs. >25 kg) (Figure 2, Table 4).

Treatment of the infection in all the patients included anti-inflammatory and combined supportive therapy. Most of them consisted of antipyretics, antibiotics, antifungals, antivirals, immunoglobulins, red blood cells, and platelet concentrates. Some patients also received albumin solutions, fresh frozen plasma, beta-mimetics and steroids in inhalation, analgesics, hepatoprotective medications, glucocorticosteroids, and pentamidine. The general condition of some patients required the implementation of oxygen therapy, forced diuresis, cardiovascular support with pressor amines, nasogastric tube feeding, or parenteral hyperalimentation. One patient underwent a lung lobectomy due to aspergilosis. Hospitalisation in the ICU was required for 21 (35.6%) patients. In 33 patients (56.9%), G-CSF was administered simultaneously with GC. Thirteen (22%) patients, after a period of improvement, received GC repeatedly due to the development of another infection accompanying neutropenia. The median time to the next administration of GC was 66 days (range: 14–427 days).

**Figure 3:**
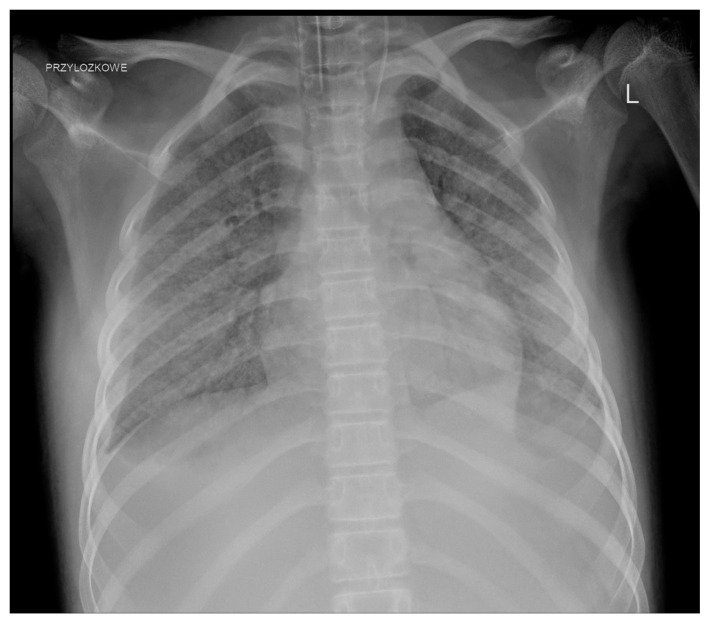
TRALI after GC transfusion.

We observed a few complications related to GC administration. One patient developed an anaphylactic reaction during the second transfusion. One girl, who required hospitalisation in the ICU as a result of infection, about 2 h after the administration of the GC, developed shock symptoms with the deterioration of the general condition and circulatory and respiratory instability; transfusion-related acute lung injury (TRALI) was diagnosed after further investigation (Figure 3).

After the fifth GC transfusion (totalling 4.5 units), alloimmunisation was observed in a male patient, which significantly complicated the selection of the subsequent blood products, particularly platelet concentrates needed for further oncological treatment (the requirement for HLA-compatible transfusions to achieve efficacy). In seven patients, after transfusion, CMV infection was diagnosed at a later stage of treatment, but a direct connection to GC transfusion remains unclear and difficult to prove.

Among the patients included in the analysis, 47 (78.33%) survived the infection which was the justification for the administration of GC. Thirteen children out of 60 (21.67%) have died. Deaths recorded in the population were attributed to the infections that were the indication for GC use, along with the associated systemic complications. The causes of death included respiratory, circulatory, or multiple organ failure; respiratory or gastrointestinal bleeding; invasive pulmonary aspergillosis; infection of CMV aetiology (CNS—1 patient; multiple organ infection—1 patient); COVID-19; sudden cardiac arrest in the course of electrolyte disturbances; metabolic acidosis; and septic shock. The median survival in this group was 23 days (IQR = 7–58). There were no statistically significant differences in the number of granulocytes/kg/day transfused to the patients who died and those who survived infection, but the groups differed significantly in the total number of granulocytes in GCs, the number of GC administrations, and the volume of transfused GCs (Table 5).

## 4. Discussion

The role of GC in patients with infection and neutropenia is well captured by researchers who describe it as a bridge to the spontaneous recovery of neutrophil counts. Properly prepared and immediately administered granulocytes have anti-inflammatory properties, both bacterial and fungal [10,11,12,17]. As well described in the paediatric literature on GC use also in our study, we showed the efficient use of GCs for the therapy of severe infection in neutropenic patients with malignant and non-malignant diseases. Over forty-eight children (78.33%) were successfully cured of the infection. A limited number of therapy-related side effects was observed in our patients.

The history of GC transfusion dates back to the mid-20th century. The first concentrates were obtained by centrifuging whole blood. Despite the introduction of dexamethasone/prednisone stimulation, the number of granulocytes obtained from healthy donors was still not satisfactory. This significant issue was only resolved with the introduction of recombinant cytokines, such as granulocyte colony-stimulating factor (G-CSF) and granulocyte-macrophage colony-stimulating factor (GM-CSF) [6,7,9,10,12].

In the majority of GC studies, donor stimulation involved the administration of both G-CSF and oral dexamethasone. G-CSF was administered in various doses (300 µg, 480 µg, 600 µg, or 5 µg/kg body weight) [7,11,18,19,20,21,22]. In some centres, a single dose of a proton pump inhibitor was added to the steroid regimen [17]. Cugno et al. summarised the research on neutrophil mobilisation methods. They found that a uniform dose of G-CSF of 480 μg is equally effective as weight-based dosing at 5 µg/kg, whereas the addition of a steroid to G-CSF provides the highest yield and is not associated with increased toxicity. It was also shown that adverse effects after G-CSF administration are usually mild and dose-related, and a high dose of G-CSF (per kilogramme of donor body weight), compared to a low dose of G-CSF combined with dexamethasone, may be associated with poorer tolerance without improved stimulation effect [11]. According to the literature, the adverse effects observed in donors after G-CSF supply included arthralgia, myalgia, headache, fatigue, and nausea. Multiple stimulations did not cause side effects exceeding WHO grade II status. Leukapheresis was also well tolerated [11,12,20]. Although a causal relationship cannot be demonstrated, Bennett et al. describe 5 cases (among 738 donors) who developed a haematologic malignancy years after receiving G-CSF [23]. This highlights the importance of long-term follow-up. In our study cohort, the donors received only G-CSF in a sustained dose of 480 µg administered subcutaneously. Unfortunately, data on adverse reactions among our donors were not collected. Based on data from NMDP and EBMT studies, the recent procedures of cell collection from donors seem to be safe both in short and long follow-up [24].

We did not observe statistically significant differences in survival depending on the aetiology of the infection. The available studies also do not provide conclusive findings in this area. Some suggest that GC is more effective in bacterial than fungal infections [25], while others report worse outcomes in patients with Gram-positive bacteraemia [26] or the lowest survival rates in infections caused by fungi and Gram-negative bacteria [19]. Still, other researchers have found no significant association between infection aetiology and clinical response [22]. However, it should be noted that due to small cohort sizes, subgroup analysis was possible only in selected studies.

The effect of GC administration has also been evaluated by other investigators. Some of them focused on clinical efficacy (resolution of infection symptoms), and radiological, microbiological, or haematological response (increase in neutrophil count) without providing information on survival. Others distinguished between infection-related survival and/or overall survival (OS) measured at different time points counted from the applied intervention or from the onset of infection (28 days, 30 days, 1 month, 90 days, 3 months, 100 days, and 180 days) [9,11,17,18,19,20,21,22,27,28]. In our study, the survival time from GC treatment to the last follow-up obtained values of 76.67% and 18 months, respectively (IQR = 2–52.5).

The efficacy of GC in studies involving predominantly adult patients was summarised by Szumowski et al., revealing a wide variation in outcomes in articles published between 2011 and 2018 [9]. These studies report diverse results in infection control and survival rates, influenced by factors such as dosage and patient characteristics. While some studies demonstrate moderate success and low infection-related mortality [29,30], others show higher mortality rates, particularly in non-standard dosing groups [31].

Cugno et al. conducted a similar summary for children, considering publications from 2003 to 2014, including both prospective and retrospective studies, with population sizes ranging from 3 to 49 patients [11].

Many studies involved small populations. In larger cohorts, Sachs et al. reported the highest infection resolution rate (92.6%) and a 30-day OS of 81.5% without showing differences in terms of infection control between the group of patients with a specific site of infection and/or documented pathogen and the group of patients with fever of unknown origin [32]. The important findings of Weingarten et al. included the observation of higher survival rates in children with lower body weight and those who received GC with higher granulocyte counts/kg body weight (i.e., above 0.13 × 10^10^) [28]. Aktekin et al. reported a clinical and haematologic response rate of 69.2% without any adverse events [21]. Kagizmanli et al. were able to retrospectively evaluate the largest group of children (74 patients) treated with GC with the following outcomes of the intervention: 1-month and 3-month survival rates of 87.8% and 76.5%, respectively [22]. Koc et al. presented very good results from GC treatment. In nine patients with 11 episodes of neutropenic fever, the clinical response rate was 90.9% and the mortality on day 30 was 9%. In this study, the early use of GCs was considered the main reason for their effectiveness [17]. The timing of GC administration appears to be a crucial factor in its efficacy; however, in most studies, GCs were often administered relatively late, usually when a patient who remains neutropenic has a prolonged infection that does not respond to standard management. It was shown that the early (i.e., <4 weeks from the onset of infection) initiation of GC transfusions leads to better responses [10]. It is suggested to use it even earlier, as delay in this intervention, when previous antimicrobial treatments fail, significantly reduces the chance of therapeutic success [7,10,11,17,20,32,33].

In our population, the median time to GC administration was also short (5 days), however comparison with other studies can be difficult. Data on the real onset of infection may be biased due to inconsistencies in its definition and description. The onset of infection could be described as increased inflammatory parameters, clinical deterioration, initiation of antimicrobial therapy, collection of material for microbiological testing, or a specific combination of all of these factors.

Delays in the beginning of GC transfusions can be explained by a lack of guidelines supported by high-quality medical research results, organisationally more difficult acquisition compared to other blood products, or, finally, a lack of methods that would allow monitoring their effect in real time. Due to the migration of transfused granulocytes to inflamed tissues, a discrepancy between clinical and haematological improvement can be observed [9,10,12]. The median time for granulocyte count to increase to more than 1000/μL since the first day of GC administration was 5 days (range 1–97) in our cohort. Granulocyte transfusions led to an increase in neutrophil count and consequently shortened the duration of neutropenia; however, most of our patients received G-CSF simultaneously (56.9% of patients). Importantly, in the therapy of serious life-threatening infections, GC is only one element of a highly combined treatment. Therefore, it is difficult to assess their effectiveness in general. This is even more difficult when the aetiology of the infection is not established. In our study, we were unable to confirm the aetiology of severe infection in 17 (27.42%) patients.

A non-negligible issue is the side effects. Initially, side effects, mainly from the respiratory system, led to the suspension of GC use [6]. The significant development of serological methods has significantly improved the safety of GC use. The most common adverse reactions are not markedly different and do not occur more frequently than during the transfusion of other blood products [6]. Some of them can be avoided by proper donor screening, appropriate pretransfusion premedication (such as antihistamines, acetaminophen, and steroids), and maintaining adequate intervals between the administration of other medications (e.g., amphotericin B) [17]. Gea-Banacloche summarised the most common toxicities, including fever, HLA sensitisation, pulmonary reactions, and CMV infection (if CMV-positive donors are used) [10]. Fever appears to be the most common complication. Alloimmunisation seems particularly problematic in the context of further anticancer treatment (in one patient, we struggled for a long time with the consequences of this complication). The risk of transfusion-transmitted infection is low as donors are routinely screened for infectious diseases, but it still exists [9]. Pulmonary complications, which are considered acute transfusion-related adverse events, appear to cause particular (and justified) concerns. Grigull et al. reported the need for the temporary escalation of respiratory support in patients undergoing mechanical ventilation, worsening of pulmonary symptoms in two children with viral pneumonia, and even the development of progressive respiratory failure during GC transfusion [18]. In a systematic review covering studies from 1966 to 2006 on granulocyte transfusions in neutropenic children, the authors observed pulmonary complications in only seven recipients, ranging from mild to more severe respiratory symptoms, but TRALI was not reported. They concluded that novel methods of leukapheresis have significantly reduced the risk of severe pulmonary complications that were observed in the past [19]. However, such reports still appear even in significantly more recent studies. Díaz et al. in a group of 13 patients with underlying acute infection observed respiratory symptoms in 6 patients (46%) including hypoxemia and tachypnea that were likely secondary to GC transfusions [34]. Weingarten et al. described one episode of life-threatening grade 4 dyspnoea (in a cohort of 21 patients) [28]. TRALI, which was diagnosed in one of our patients, remained in close association with GC administration.

In our group, the incidence of presented toxicities after GC transfusions (5%) was similar to that in other studies. According to the literature, more serious toxicities affect about 5% of the recipients [6]. Although some scientists have not reported adverse events associated with GC administration [17,21], most researchers have noted their appearance [9,10,11,18,27]. Serious post-transfusion reactions we described in our three patients seem to have an autoimmune basis. Therefore, they are in some way independent of technological advancements.

Although there were no statistically significant differences in the number of transfused granulocytes/kg/day between the patients who survived and those who died, most neutropenic patients were cured of severe infections (78.33%).

Notably, the patients who died received significantly greater total GC granulocyte counts, number of GC administrations, and volume of GCs transfused compared with those who survived. This may be explained by the severe general condition of these patients and the increased need for GC transfusions.

In the literature, the most commonly postulated value for expecting a satisfactory clinical effect was 1.5 × 10^8^ granulocytes per kilogramme of body weight, but the term high-dose granulocyte transfusion was reserved for 0.6 × 10^9^ per kilogramme of body weight [10,16]. The optimal dose of granulocytes was more feasible in younger recipients and therefore with lower body weight [28], as was also shown in our study. A sufficient dose of granulocytes for infusion may be an issue in older children and adult patients.

Finally, it is important to note the reasons for the poor quality of the evidence on GC transfusion and the difficulties in drawing conclusions about the justification for implementing this procedure [7,9]. One of the limitations of our study is its retrospective nature and the lack of control groups and randomisation. Moreover, in 25% of the cases, the cause of the infection could not be identified. The lack of follow-up data on donor outcomes, limited access to detailed GC data for only a subset of patients, and subjective clinical judgments regarding the timing of GC administration and duration of the intervention also affect the quality of our results.

## 5. Conclusions

Based on literature data and our study, it appears that GC may be beneficial for neutropenic children with severe infections and could potentially help reduce infection-related mortality. The early initiation of GC transfusion may, but does not necessarily, results in better clinical responses, but it is worth considering in patients with infection accompanying neutropenia and no response to standard management. Granulocytes should be administered in sufficient numbers (calculated per kilogramme of body weight) and have the ability to migrate to sites affected by inflammation. The granulocyte concentrate obtained by apheresis appears to contain cells that meet these requirements. Granulocyte counts in the concentrate considered sufficient are easier to achieve in younger patients (and therefore with lower body weights)—in older children, more frequent quality controls may be warranted. Patients receiving GC must be closely monitored for adverse reactions. In the context of donors, attention should also be paid not only to the efficacy of stimulation but also to potential short- and long-term side effects.

The nature of our study, along with its limitations, precludes us from making definitive recommendations regarding GC transfusion in the specific cohort and indication. It seems that only a well-conducted, preferably multicentre randomized controlled trial could serve as the basis for the development of new guidelines in this area.

## Figures and Tables

**Table 1:** The characteristics of the patients.

Variables	Frequency (Percentage)
Number of patients	60
Sex (male %)	33 (55.0)
Age (median; range)	9.5 years (4 months–19 years)
Weight [kg] (median; IQR)	30.75 (18.78–32.0)
**Diagnosis**	
Acute myeloid leukaemia (%)	22 (36.67)
Acute lymphoblastic leukaemia (%)	19 (31.67)
Other leukaemias (MPAL/early T-II, JMML) (%)	1 (1.67)
Lymphomas (%)	6 (10)
Other oncological diseases (%)	8 (13.3)
Non-oncological diseases (%)	4 (6.67)
**Patients’ disease status**	
First complete remission (%)	38 (63.33)
Relapse (%)	13 (21.67)
Secondary cancer (%)	5 (8.33)

**Table 2:** The details of the infections.

Variables	Number	(%)
**Aetiology of the infections**		
Bacterial	23	38.33
Fungal	12	20.00
Viral	3	5.00
Unidentified	15	25.00
More than one pathogen	5	8.33
**Investigations leading to the diagnosis**		
Blood culture	23	38.33
Fungal serology	4	6.67
Histopathology (biopsy of the lesion)	2	3.33
Imaging studies (chest X-ray/lung ultrasonography/CT scan)	15	25.00
Wound smear	4	6.67
Stool examination	6	10.00
Other material (peritoneal fluid, nasopharyngeal swab, bronchoalveolar lavage, tracheostomy tube swab, tracheal aspirate, or sputum)	7	11.67
More than one diagnostic test leading to identification	12	20.00

**Table 3:** Transfusion data and lab test results.

Variables	Min.	Max	Median	Q1	Q3
Time until initiation of GC treatment [days]	1	39	5	3	9
Number of GC administrations	1	29	2	1	3
Duration of GC administration [days]	1	70	3	1	5.5
Total volume of transfused GC [mL]	200	9190	656.5	400	1223.25
Time to reach granulocyte count >1000/μL [days]	1	97	5	2	9
Leukocyte count at the time of GC initiation [/μL]	0	1340	180	100	460
CRP max [mg/L]	58.1	770.6	260.9	200.4	375.1
PCT max [ng/mL]	0.1	129.8	2.7	0.6	21.7

**Table 4:** Number and percentage of GCs meeting the quality standards depending on the patient’s weight.

Variables	Body Weight ≤ 25 [kg]	Body Weight > 25 [kg]
Number of GC	57	86
≥1.5 × 10^8^/kg	55 (96%)	54 (63%)
≥6 × 10^8^/kg	25 (44%)	10 (12%)

**Table 5:** Comparison of survival and non-survival groups.

Variables	Survival Group	Non-Survival Group	*p*-Value
Number of patients	47 (78.33%)	13 (21.67%)	
Number of granulocytes/kg/day [×10^8^]	2.5 (IQR 1.4–3.8)	2.3 (IQR 1.8–3.1)	0.41
Total number of granulocytes in transfused GC [×10^10^]	1.9 (IQR 1.4–1.3)	5.2 (IQR 2.5–6,5)	**0.04**
Number of GC administrations	2.0 (IQR 1.0–3.0)	3.0 (IQR 2.0–6.0)	**0.02**

Bold in the *p*-value column indicates statistical significance.

## Data Availability

The data are not publicly available due to privacy and ethical restrictions.
